# Renal Tissue-Derived Exosomal miRNA-34a in Diabetic Nephropathy Induces Renal Tubular Cell Fibrosis by Promoting the Polarization of M1 Macrophages

**DOI:** 10.1049/2024/5702517

**Published:** 2024-04-17

**Authors:** Shuai Zheng, Yi Zeng, Liqing Chu, Taiyang Gong, Sihong Li, Min Yang

**Affiliations:** Department of Nephrology, The Second Affiliated Hospital, Kunming Medical University, No. 347 Dianmian Street, Kunming, Yunnan 650101, China

## Abstract

**Background:**

Diabetic nephropathy (DN) is the leading cause of chronic kidney disease, and the activation and infiltration of phagocytes are critical steps of DN. This study aimed to explore the mechanism of exosomes in macrophages and diabetes nephropathy and the role of miRNA-34a, which might provide a new path for treating DN.

**Materials and Methods:**

The DN model was established, and the success of the model establishment was confirmed by detecting general indicators, HE staining, and immunohistochemistry. Electron microscopy and NanoSight Tracking Analysis (NTA) were used to see the morphology and size of exosomes. MiRNA-34a inhibitor, miRNA-34a mimics, pc-*PPARGC1A*, and controls were transfected in macrophages with or without kidney exosomal. A dual-luciferase reporter gene experiment verifies the targeting relationship between miRNA-34a and *PPARGC1A*. After exosomal culture, macrophages are co-cultured with normal renal tubular cells to detect renal tubular cell fibrosis. Q-PCR and western blot were undertaken to detect related RNA and proteins.

**Results:**

An animal model of diabetic nephropathy was successfully constructed. Macrophages could phagocytose exosomes. After ingesting model exosomes, M1 macrophages were activated, while M2 macrophages were weakened, indicating the model mice's kidney exosomes caused the polarization. MiRNA-34a inhibitor increased *PPARGC1A* expression. MiRNA-34a expressed higher in diabetic nephropathy Model-Exo. MiRNA-34a negatively regulated *PPARGC1A*. *PPARGC1A* rescued macrophage polarization and renal tubular cell fibrosis.

**Conclusion:**

Exosomal miRNA-34a of tubular epithelial cells promoted M1 macrophage activation in diabetic nephropathy via negatively regulating *PPARGC1A* expression, which may provide a new direction for further exploration of DN treatment.

## 1. Introduction

Diabetic nephropathy (DN) is the changes in chronic kidney structure and dysfunction caused by diabetes, which has become the leading cause of chronic kidney disease and end-stage renal disease [1]. According to statistics from the International Diabetes Federation (IDF), the number of diabetes patients worldwide in 2015 was 415 million, and it is expected to increase to 552 million in 2035 [2]. Pathological changes related to DN include macrophage infiltration, glomerular sclerosis and atrophy, interstitial fibrosis, and loss of renal function [3]. More evidence indicates that the renal tubules play a more critical role in the development of DN [4].

Due to its complex metabolism disorders, diabetic nephropathy is often more tricky than the treatment of other kidney diseases [5]. An in-depth understanding of the pathogenesis of DN and intervention of the critical links of the disease reduced the pain caused by the disease. At present, the pathogenesis of DN is still not fully clear. Previous studies have suggested that regulating miRNA in the gene may become a new direction for DN treatment [6, 7]. It is worth noting that exosomal miRNA may have potential research value in the development of diabetic nephropathy. Xie [8] reported that 391 miRNA expressions in urine exosomes of type 2 diabetic nephropathy were different from those of type 2 diabetic patients. The severity of tubular interstitial lesions in DN is closely related to the progressive decline of proteinuria excretion and renal function, suggesting renal tubular epithelial cell (TEC) damage is very important in DN [9]. Interestingly, miRNA-34a was confirmed to participate in glomerular hypertrophy progress, further accelerating the early DN.

Importantly, miR-34A was confirmed to play a vital role in the response reaction of macrophages [10, 11]. Macrophages are a type of innate immune cell that have the functions of chemotaxis, phagocytosis, inflammation regulation, and killing microorganisms [12]. They can also ingest, process, and present antigens to T cells for recognition and participate in specific immune responses [13]. Among them, M1 macrophages are characterized by secreting a large number of inflammatory factors, producing inducible nitric oxide synthase (iNOS), reactive oxygen intermediates (ROI), reactive nitrogen intermediates (RNI), and enhancing the ability of antigen presentation [14]. It is the primary effector cell for the host to destroy pathogens, but continuous M1 activation and its reaction products may also cause tissue damage. Research using gene-deficient mice (ICAM-deficient and MCP-1-deficient) to reduce kidney damage in db/db mice strongly suggests that macrophages are critical in DN immune damage [15]. Notably, both clinical and animal experiments confirm the presence of M1-type macrophages in the diabetic tissue injury site. Then, does miRNA-34a in renal tubular epithelial cell exosomes participate in the immune process of macrophages in DN?

In this study, we established a DN model; transfected the miRNA-34a inhibitor, miRNA-34a mimics, pc-PPARGC1A, and empty control in macrophages with or without exosomes; cocultured macrophages with normal renal tubular cells; and detected renal tubular cell fibrosis. We verified the targeting relationship between miRNA-34a and PPARGC1A using a dual-luciferase reporter gene assay. We detected related RNA and proteins to explore the role and mechanism of miRNA-34a in the immune process of macrophages in DN, providing effective potential targets for the treatment of DN.

## 2. Materials and Methods

### 2.1. Construction of the Animal Model

A total of 10 SPF C57BL/KsJ db/db mice [16] (4 weeks old, male, 20.13 ± 1.28 g) were selected, and 10 db/m mice born in the same litter (4 weeks old, male, 13.92 ± 0.69 g) were included as control. All animals were purchased from the Institute of Model Animals of Nanjing University. The mice were fed in the animal experiment center of our hospital and provided generally for a week to adapt to the new environment. The mice eat and drink freely, keeping the atmosphere clean and dry. The feeding temperature was 25°C, the relative humidity was 60%–80%, and the day and night were alternately illuminated for 12 hr.

The body weights of both the db/db mice and db/m mice groups were assessed weekly. Additionally, tail-tip venous blood samples were collected biweekly, followed by the centrifugation of the supernatant. The levels of blood glucose, blood urea nitrogen (BUN), and serum creatinine (Scr) were then measured using an automatic biochemistry analyzer (Hitachi, Tokyo, Japan) specifically designed for mice. Urine was collected using a metabolic cage. The uric acid (UA) detection kit (C012-1-1, Jiancheng Biotechnology, Nanjing, China) was used to determine UA content when the above indicators of db/db mice were significantly different from those of db/m mice (*P* < 0.05). It indicated that the animal model of diabetic nephropathy was successfully constructed.

### 2.2. Macrophage Culture and Treatment

Macrophages (RAW264.7) [17] were purchased from the Shanghai Chinese Academy of Sciences Cell Bank. RAW264.7 cells were seeded in a 100-mm diameter dish at 1 × 10^6^/mL and cultured in DMEM containing 10% fetal bovine serum at 37°C and 5% CO_2_.

One hundred microliter of kidney exosomal fluid at a 100 *μ*g/mL concentration was resuspended in 1 mL PBS, and then 4 *μ*L PKH26 fluorescent dye solution was added. After incubating at 37°C for 20 min, the mixture was centrifuged at 100,000 × *g* for 70 min at 4°C, the supernatant was discarded, and the exosomes were gently resuspended in 10 mL PBS. After centrifugation at 100,000 × *g* for 70 min at 4°C, the excess dye was removed, the supernatant was discarded, and the exosomes were resuspended in 100 *μ*L PBS for later use. RAW264.7 cells were resuspended in a serum-free medium and placed in a 37°C, 5% CO_2_ incubator. After the cells adhered to the wall, PKH26 fluorescently labeled exosomes were added. After 12 hr of incubation, the cells were washed twice with PBS, fixed with 4% paraformaldehyde, and stained with DAPI. A confocal fluorescence microscope was used to observe whether exosomes enter the cell. PKH26 fluorescently labeled exosomes showed red fluorescence under the microscope.

For transfection, miRNA-34a inhibitor, inhibitor control, miRNA-34a mimics, mimics control, pc-*PPARGC1A*, and pc-NC were all designed and synthesized in Biomics Biotechnology Co., Ltd., (China) (please refer to *Supplementary 2* (Sequence) for more information). The transfection reagent Lipofectamine 2000 (11668-027, Invitrogen). After trypsinization of RAW264.7 cells, PBS was added to wash the cells twice, and an appropriate amount of cell growth medium was added to adjust the cell concentration to 3 × 10^5^ cells/mL. When the cell fusion reached 60%, the cell growth medium was discarded, and the incomplete cell growth medium without FBS was added and placed in a 37°C, 5% CO_2_ incubator for 1 hr. MiRNA-34a inhibitor and inhibitor control were added to the incomplete medium and mixed thoroughly to prepare liquid A. Serum-free incomplete medium was mixed with transfection reagent Lipofectamine 2000 to prepare B solution. Appropriate amounts of liquids A and B are mixed and placed at room temperature for 20 min. The cell culture medium was discarded, PBS was used for washing, and the mixture was added to the cells. After being placed in a 37°C, 5% CO_2_ incubator for 6 hr, the complete medium was replaced, and the culture was continued for 48 hr.

### 2.3. Coculture of Macrophages with Exosome Uptake and Normal Renal Tubular Cells

Renal tubular epithelial cells of the mouse (TCMK-1 cell line) were also purchased from the Shanghai Chinese Academy of Sciences Cell Bank. The renal tubular epithelial cells were seeded in a 96-well culture plate at a density of 5 × 10^3^/cm^2^. When the cells grow to about 50%–60%, resuspend them in 300 *μ*L of 10% FBS DMEM medium, and put them into the macrophage two-cell culture system after exosome culture and further culture for 24 hr [17].

### 2.4. HE Staining

The mice were anesthetized with 1% pentobarbital sodium, and blood was taken from the heart. The kidneys were quickly dissected and unencapsulated; half of them were frozen in liquid nitrogen, and the other half were fixed in 4% paraformaldehyde for paraffin embedding. Paraffin sections were prepared according to the usual method. After baking the slices at 65°C for 1.5 hr, the gradient was deparaffinized and rehydrated, and the HE staining was strictly in accordance with the instructions of the kit.

### 2.5. Immunohistochemistry Assay

In this study, the horseradish peroxidase-labeled antibody method was used for immunohistochemical samples. The sections were deparaffinized, dehydrated by alcohol gradient and antigen retrieval, and then rinsed with 0.01 mol/L PBST. Then, the samples were immersed in 2% BSA and sealed in a humidified box at 37°C for 30 min. After that, the F4/80 primary antibody (ab300421, Abcam, 1 : 5,000) was dropped onto the sample and incubated at 37°C for 1 hr. After rinsing, 1 : 500 horseradish peroxidase secondary antibody (ab150081, Abcam) was added dropwise and incubated for 60 min. Finally, the sample was rinsed and mounted, observed, and photographed under a light microscope.

### 2.6. Extraction of Kidney Exosomes from Model Mice

The kidney tissue was chopped to a homogenate shape, and the homogenate was placed in a test tube containing 75 U/mL type 3 collagenase and Hibernate-E. A total of 800 *μ*L of collagenase was added to every 100 mg of tissue. The tissue was incubated in a 37°C water bath shaker for 30 min. During this period, the tube was inverted and mixed once every 5 min and pipetted twice with a pipette. Exosomes were separated from the harvested supernatant according to the previous study [18]. Tissue cells were removed by centrifugation at 300 × *g* for 10 min at 4°C, and then the sample was centrifuged at 2,000 × *g* for 20 min to remove debris. The large vesicles were centrifuged at 10,000 × *g* for 30 min to remove them, and the supernatant was removed by ultracentrifugation at 140,000 × *g* for 90 min. The obtained pellet was exosomes. After the pellet was washed and resuspended in PBS buffer, it was centrifuged at 140,000 × *g* for 90 min, and the exocrine body was suspended in PBS, filtered with a 0.22-*μ*m pore filter, and stored at −80°C. The BCA method was used to determine the concentration of exosomes.

### 2.7. Electron Microscopy Analysis

A total of 1 *μ*L of the above solution was adsorbed on a 200-mesh carbon support film, fixed with 1% hydronium for 10 min, washed with ultrapure water for 5 min, and observed and collected images under an HT7700 transmission electron microscope.

### 2.8. Nanoparticle Tracer Analysis

Nanoparticle tracer analysis was processed by collecting and observing the scattered light signals of nanoparticles in real time through an optical microscope and then capturing the Brownian motion of the nanoparticles in the solution. Finally, each Brownian motion particle was tracked and analyzed, and the hydrodynamic radius and concentration of the nanoparticle were quickly and accurately calculated. The instrument used was Malvern Nanosight NS300, nano3.4 system; the particle size range of detection is usually 10 – 2,000 nm, and the sample concentration range is 10^7^ – 10^9^ PCS/mL. Sample loading after startup and cleaning, the mother liquor of exosomes was diluted with water to 10 mL at a ratio of 1 : 7,500; the instrument parameters were set; the details of parameter Settings are as follows: Screen Gain, 4; Camera Level, 11; Select Dilution for Standard Measurement, 7,500; focus, 182; capture duration, 50 s; and Detection Threshold, 5. Then adjust the field of view and start the detection, and save the result after the instrument automatically analyzes the result and calculatethe concentration of exosomes in the sample.

### 2.9. Western Blot Assay

Total cells of all groups were harvested, and the protein concentration of exosomes was determined with the BCA Protein Quantitative Detection Kit. The protein expression in cocultured cells was also processed as follows. After calculating the sample and loading buffer volume according to the measured protein concentration, the sample is mixed and placed in a water bath at 95°C for 5 min. A 10% SDS-polyacrylamide gel (SDS–PAGE) was prepared, the protein concentration was diluted to 5 *μ*g/*μ*L before loading and 10 *μ*L/well at the time of loading; the protein was separated by electrophoresis, and then the protein was transferred to the PVDF membrane at 200 mA. After blocking for 2 hr, they were incubated overnight at 4°C with primary antibodies (TSG101, ab125011; Grp94, ab238126; CD63, ab134045; Arg1, ab96183; iNOS, ab178945; CD86, ab239075; CD206, ab125028; FN, ab268020; *α*-SMA, ab124964; Collagen I, ab34710, 1 : 1,000, Abcam). TBST was used to wash the membrane for three times. After the chemiluminescent agent develops the color, the protein gel imaging system analyzes the relative content of the target protein in the sample. The experiment was repeated three times.

### 2.10. Q-PCR Assay

Q-PCR method was undertaken to detect the expression of miRNA-34a, iNOS, CD86, CD206, and Arg-1 in all groups. Reagents, including triazole lysate, Premix ExTaq version 2.0, and SYBR PremixEx Taq, were purchased from Takara (Japan). The PCR primers were synthesized by Shanghai Sangon Biotech Company. A fluorescence PCR instrument was used to detect the relative expression of miRNA-34a in cDNA. Reagents were added to the sample in sequence; the final total volume was 20 *μ*L. The reaction conditions were 95°C pre-denaturation for 10 min, 95°C for 15 s, 60°C for 15 s, 45 cycles, and a fluorescence signal temperature of 60°C. U6 and *ß*-actin were the internal references, and the q-PCR assay was performed using the 2^−*ΔΔ*Ct^ method. The expression of *PPARGC1A* in macrophages with model exosomes was also detected by the above steps.

### 2.11. Immunofluorescence Assay

Cells of each group were planted on a cover glass and then treated with Model-Exo, Ctrl-ExoH, Model-Exo+pc-NC, Model-Exo+pc-*PPARGC1A*, and PBS for 6 hr after being attached, and fixed with 4% paraformaldehyde for 1 hr at room temperature. The sample was rinsed three times for 10 min with 0.01 mol/L PBS, blocking serum was added, and they were incubated at room temperature for 2 hr. After the blocking serum was removed, the primary antibody monoclonal antibody (*Arg1*, ab96183*; iNOS*, ab178945*; CD86*, ab119857*; CD206*, ab300621; *FN*, ab268020*; α-SMA*, ab124964*; Collagen I*, ab270993;*ß-actin*, ab8226, 1 : 100, Abcam) were added to the sample and incubated for 1 hr at room temperature and then incubated overnight at 4°C. After rinsing with 0.01 mol/L PBS, the secondary antibody (ab150081, ab175783, ab125900, 1 : 1,000, Abcam) mixture was added to the sample and incubated for 2 hr at 4°C in the dark. After fluorescence mounting, the sample was observed under a laser confocal microscope. Immunofluorescence assay was undertaken in cocultured cells according to the above steps.

### 2.12. Dual-Luciferase Reporter Experiment

The pmirGLO plasmid containing *PPARGC1A* wild-type (WT) 3′-UTR and the pmirGLO plasmid containing *PPARGC1A* mutant (MT) 3′-UTR were cotransfected with miRNA-34a mimic and miR-NC into HEK293 cells, respectively. Six wells were set up in each group. After transfection for 36 hr, the dual-luciferase detection kit was used to detect firefly luciferase activity (Beyotime, RG027).

### 2.13. Statistical Analysis

The data obtained in the experiment was analyzed with the software GraphPad Prism8 and presented as mean ± SD. Due to the small amount of data in this experiment, the Shapiro–Wilk test was used to test the normality of all data. When comparing the means of two relevant samples, if the difference followed the normal distribution, the unpaired *t*-test was used. If the difference did not follow the normal distribution, the Wilcoxon one-sample test was used. When comparing the means of multiple samples, one-way ANOVA was used if the variance was homogeneous, and each sample conformed to the normal distribution. The Kruskal–Wallis test was used if the variance was uneven or a sample did not follow the normal distribution. *P* < 0.05 indicated that the difference was statistically significant. If the results were compelling, multiple comparisons were made, such as the SNK method, LSD method, and extended *t*-test.

## 3. Results

### 3.1. The Animal Model of Diabetic Nephropathy Was Successfully Constructed

**Figure 1:**
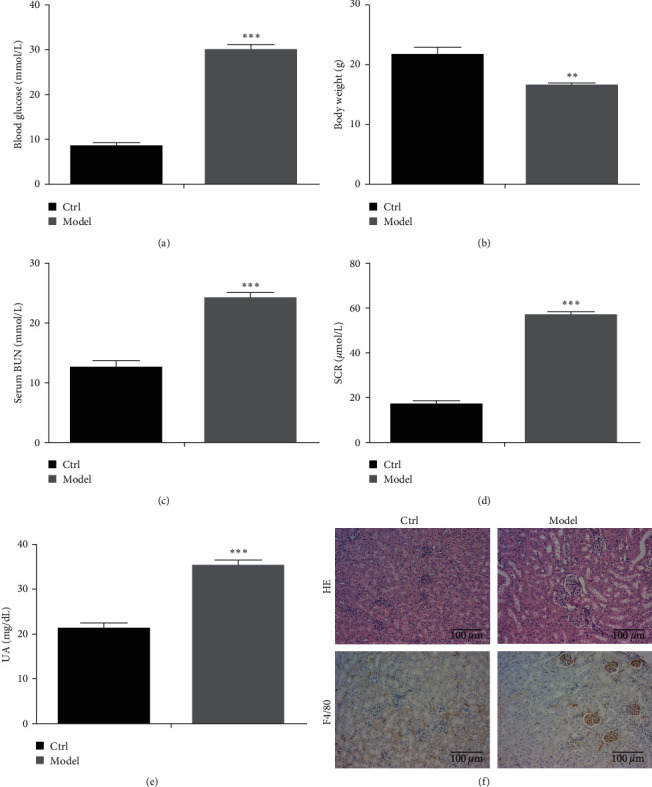
A mouse model of diabetic nephropathy was successfully constructed: (a) blood glucose concentration, (b) the weight of the mouse in the model and control groups, (c) BUN concentration in serum, (d) Scr concentration in serum, (e) content of urine uric acid, and (f) HE staining and F4/80 immunohistochemical assay.  ^*∗∗*^*P* < 0.01,  ^*∗∗∗*^*P* < 0.001*vs*. Ctrl group.

After the diabetic nephropathy model was constructed, fundamental physical indicators of mice were measured. The blood glucose, serum BUN, and Scr concentrations were significantly increased, while there was a significant weight loss in mice in the model group compared to those in the control group (Figure 1(a)–1(d)). Furthermore, UUA concentration was obviously higher in the model group than in the controls (Figure 1(e)). The results of HE staining and F4/80 immunohistochemical assay showed that the degree of inflammation in the model group was significantly higher than that of the control group, and the amount of F4/80 was also considerably higher than that of the control group (Figure 1(f)). All these results indicated that the diabetic nephropathy model was successfully constructed.

### 3.2. Extraction and Identification of Kidney Exosomes in Model Mice and miRNA Detection

**Figure 2:**
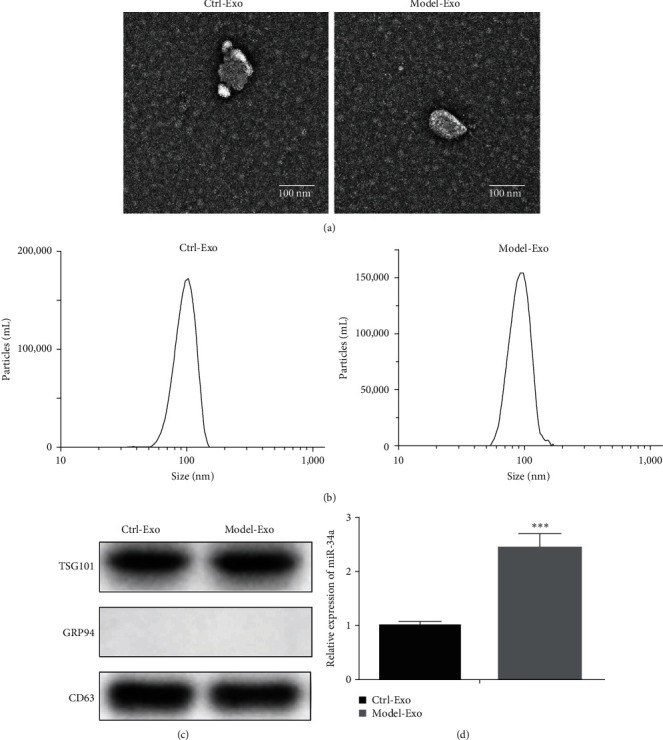
Extraction and identification of kidney exosomes in model mice and miRNA detection: (a) morphology of kidney exosomes under electron microscope, (b) nanoparticle tracking analysis of exosomes, (c) expression of exosomal markers (*TSG101*, *Grp94*, *CD63*), and (d) Q-PCR detection of miRNA-34a in kidney exosomes.  ^*∗∗∗*^*P* < 0.001 *vs*. Ctrl-Exo group.

A disk-shaped vesicle structure with a size of 40–150 nm was observed in the isolated exosomes using a transmission electron microscope (Figure 2(a)). Based on the results of the NanoSight Tracking Analysis (NTA) measurement, the particle size distribution of the sample particles was 40–150 nm, which was consistent with the size of exosomes (Figure 2(b), *Supplementary 1*). Western blot analysis showed that the collected particles contained exosomal markers such as TSG101 and CD63 and did not contain Grp94, further confirming that the isolated samples were exosomes (Figure 2(c)). Interestingly, the miRNA-34a in kidney exosomes of the model group was significantly higher than that in the control group (Figure 2(d)). Therefore, studying the mechanism of exosomes miRNA-34a is of great significance for the study of diabetic nephropathy.

### 3.3. The Kidney Exosomes of Model Mice Caused the Polarization of Macrophages

**Figure 3:**
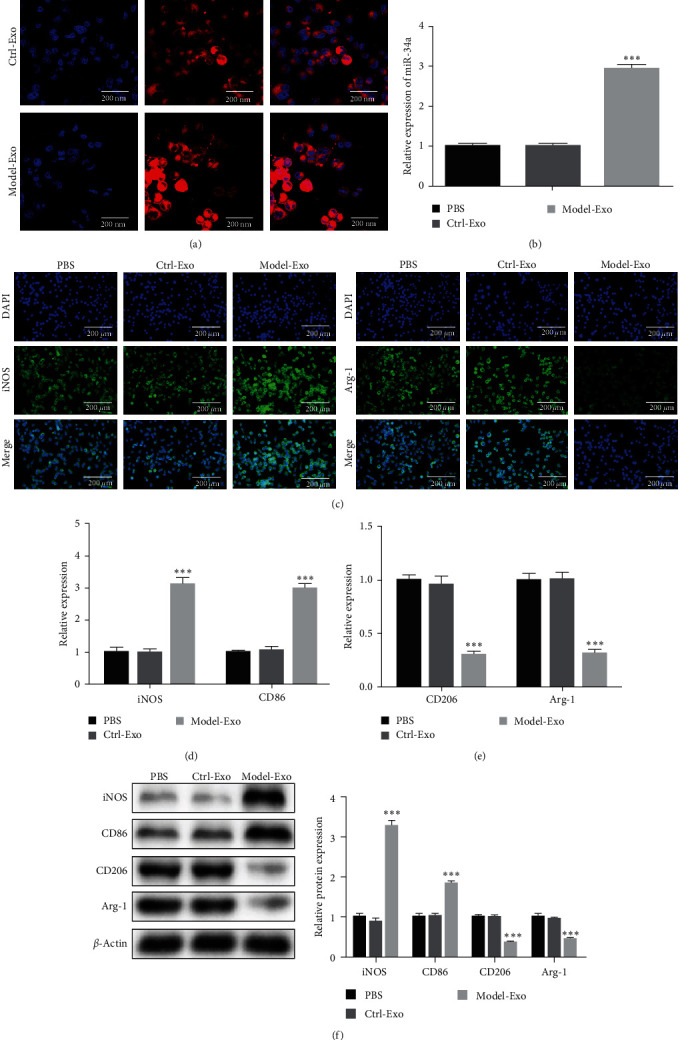
The kidney exosomes of model mice caused the polarization of macrophages: (a) macrophages take up PKH26-labeled kidney exosomes, (b) macrophage miRNA-34a expression after uptake of exosomes, (c) immunofluorescence method was processed to observe the expression of *iNOS* and *Arg1*, (d) expression of macrophage M1 phenotypic markers (*iNOS* and *CD86*), (e) expression of macrophage M2 phenotypic markers (*CD206* and *Arg-1*), and (f) western blot detection was undertaken to detect macrophage M1 and M2 phenotypic markers.  ^*∗∗∗*^*P* < 0.001*vs*. Ctrl-Exo group.

In order to research the effect of exosomes on the polarization of macrophages, the experiments were divided into three groups, including PBS, Ctrl-Exo, and Model-Exo group. In both the Ctrl-Exo and Model-Exo groups, macrophages take up PKH26-labeled kidney exosomes (Figure 3(a)). qPCR assay was undertaken to detect the expression of miRNA-34a in macrophages after the uptake of exosomes. As a result, miRNA-34a was expressed higher in the Model-Exo group than in the other two groups (Figure 3(b)). Notably, after ingesting model exosomes, M1 macrophages were activated, while M2 macrophages were weakened. In the Model-Exo group, the expression of M1 phenotypic markers (*iNOS* and *CD86*) increased, while the expression of macrophage M2 phenotypic markers (*CD206* and *Arg-1*) decreased (Figure 3(c)–3(f)).

### 3.4. Coculture of Macrophages with Model Exosomes and TCMK-1 Cells Induced Fibrosis of the Renaltubular Cells

**Figure 4:**
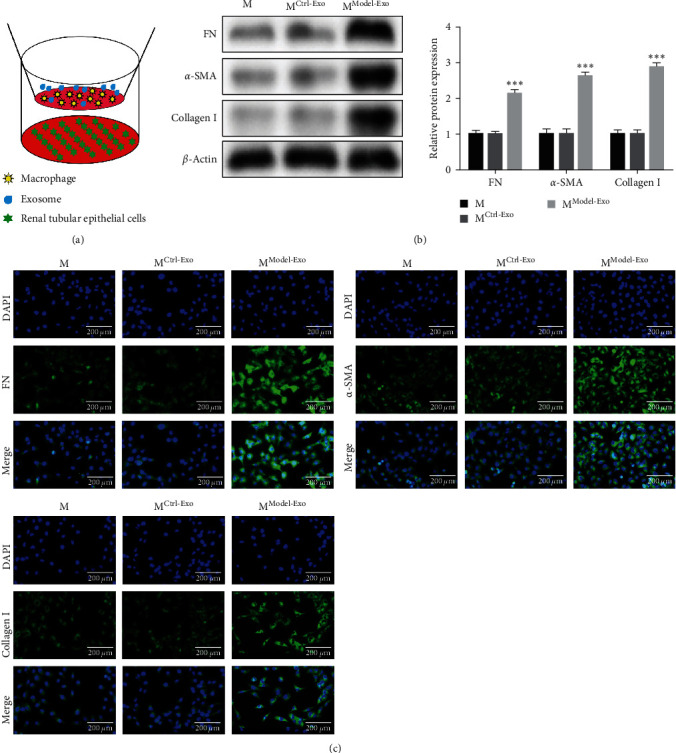
Macrophages treated with model exosomes promote renal tubular cell TCMK-1 fibrosis: (a) schematic diagram of cocultivation, (b) western blot detection for fibrosis-related indicators (FN/*α*-SMA/Collagen I), and (c) immunofluorescence detection for fibrosis-related indicators (FN/*α*-SMA/Collagen I).  ^*∗∗∗*^*P* < 0.001*vs*. M^Ctrl-Exo^ group.

The function of macrophages with model exosomes was researched, and the cells were divided into three groups, including macrophage (M), M^Ctrl-Exo^, and M^Model-Exo^ groups. Themacrophages with model exosomes and TCMK-1 cells were cocultured, and the cocultivation diagram is shown in Figure 4(a). Western blot and immunofluorescence assay were processed to detect the expression of fibrosis-related indicators (FN, *α*-SMA, and Collagen I). As shown in Figures 4(b) and 4(c), FN, *α*-SMA, and Collagen I were higher in M^Model-Exo^ than those in the other two groups. Thereby, the coculture of macrophages with model exosomes and TCMK-1 cells induced prominent fibrosis of the renal tubular cells.

### 3.5. miRNA-34a-Targeted PPARGC1A

**Figure 5:**
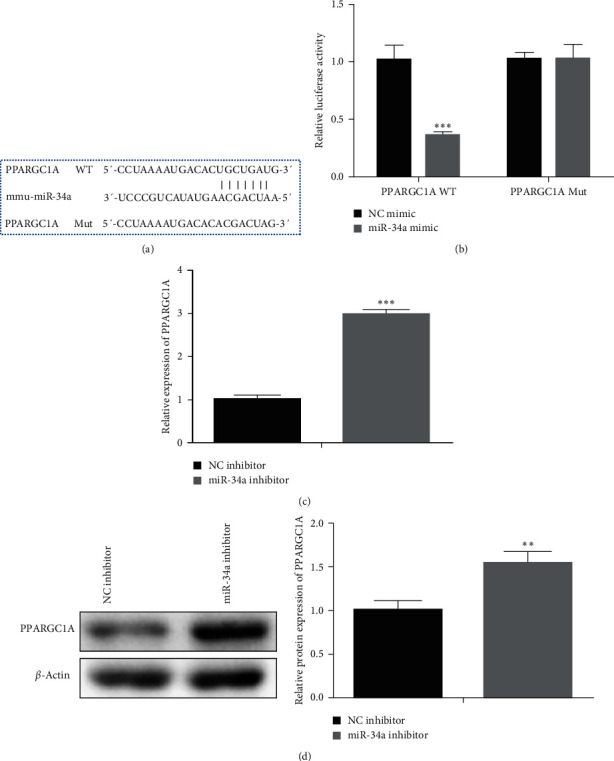
miRNA-34a-targeted *PPARGC1A*: (a) the binding site between *PPARGC1A* and miRNA-34a, (b) miRNA-34a mimic significantly decreased the expression of *PPARGC1A*, (c) miRNA-34a inhibitor obviously increased *PPARGC1A* expression, and (d) Q-PCR detection for the relationship between *PPARGC1A* and miRNA-34a expression.  ^*∗∗*^*P* < 0.01,  ^*∗∗∗*^*P* < 0.001*vs*. NC inhibitor group.

Based on the targetscan database (https://www.targetscan.org/vert_72/), *PPARGC1A* was the target of miRNA-34a. The binding sites are shown in Figure 5(a). In the *PPARGC1A* WT group, miRNA-34a mimic significantly decreased the expression of *PPARGC1A*. However, miRNA-34a mimic transfection had no effect on the *PPARGC1A* level in the *PPARGC1A* Mut group (Figure 5(b)). Q-PCR and western blot assay were undertaken to detect *PPARGC1A* expression. As shown in Figures 5(c) and 5(d), miRNA-34a inhibitor obviously increased *PPARGC1A* expression. The above results confirmed that miRNA-34a-targeted *PPARGC1A*.

### 3.6. pc-PPARGC1A Rescued Macrophage Polarization

**Figure 6:**
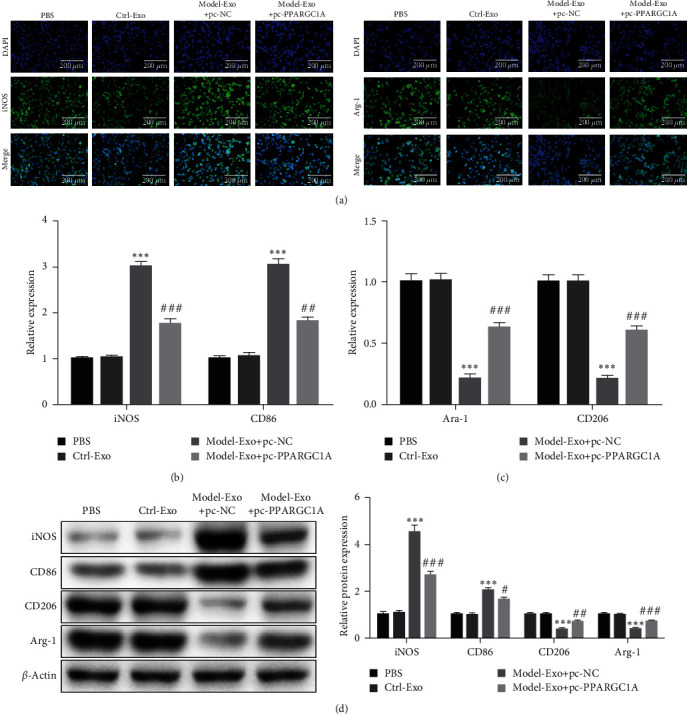
pc-*PPARGC1A* rescued macrophage polarization: (a) the immunofluorescence method was used to observe the expression of *iNOS* and *Arg1*, (b) expression of macrophage M1 phenotypic markers (*iNOS* and *CD86*), (c) expression of macrophage M2 phenotypic markers (*CD206* and *Arg-1*), and (d) western blot detection was undertaken to detect macrophage M1 and M2 phenotypic markers. Compared with Ctrl-Exo group,  ^*∗∗∗*^*P* < 0.001, compared with Model-Exo+pc-NC group, ^#^*P* < 0.05, ^##^*P* < 0.01, ^###^*P* < 0.001.

In order to study the effect of the miRNA-34a/*PPARGC1A* axis on the polarization of macrophages, four groups were divided, including Model-Exo+pc-*PPARGC1A*, Model-Exo+pc-NC, Ctrl-Exo, and PBS groups. In the Model-Exo+pc-NC group, the expression of M1 phenotypic markers (*iNOS* and *CD86*) increased, while the expression of macrophage M2 phenotypic markers (*CD206* and *Arg-1*) decreased. Interestingly, pc-*PPARGC1A* rescued macrophage polarization in the Model-Exo+pc-*PPARGC1A* group (Figure 6(a)–6(d)). The results confirmed that *PPARGC1A* recovered macrophage polarization induced by exome.

### 3.7. pc-PPARGC1A Rescued the Effect of Macrophages on Renal Tubular Cell Fibrosis

**Figure 7:**
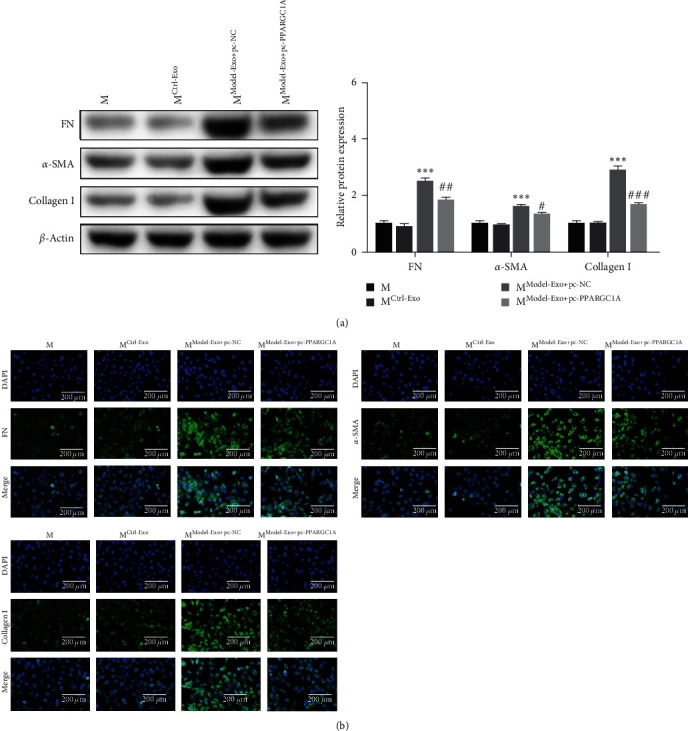
pc-*PPARGC1A* rescued the effect of macrophages on renal tubular cell fibrosis: (a) western blot detection for fibrosis-related indicators (FN/*α*-SMA/Collagen I) and (b) immunofluorescence detection for fibrosis-related indicators (FN/*α*-SMA/Collagen I). Compared with Ctrl-Exo group,  ^*∗∗∗*^*P* < 0.001, compared with Model-Eexo+pc-NC group, ^#^*P* < 0.05, ^##^*P* < 0.01, ^###^*P* < 0.001.

The effect of the miRNA-34a/*PPARGC1A* axis on renal tubular cell fibrosis was also researched in four groups, including Model-Exo+pc-*PPARGC1A*, Model-Exo+pc-NC, Ctrl-Exo, and PBS groups. FN, *α*-SMA, and Collagen I were decreased in Model-Exo+pc-*PPARGC1A* than those in Model-Exo+pc-NC (Figures 7(a) and 7(b)). Thereby, pc-*PPARGC1A* significantly rescued fibrosis of the renal tubular cells induced by model exosomes.

## 4. Discussion

Diabetic nephropathy is a common clinical microvascular complication [19]. Previous studies have confirmed that the infiltration of macrophages is an essential factor in the damage and destruction of the kidney structure [20, 21]. So, how are macrophages activated to infiltrate? In this study, we researched the mechanism of exosomal miRNA by simulating the microenvironment of diabetic nephropathy. It was confirmed that in this model, exosomes were successfully phagocytosed by macrophages, participated in the inflammatory response, and further caused renal tubular cell fibrosis.

Exosomes are necessary carriers for material and information exchange between cells [22]. They carried a variety of materials and participated in immune response, antigen presentation, cell migration, cell differentiation, and tumor invasion [23]. Previous studies have reported that exosomes have a critical role in the pathogenesis of diabetic nephropathy [24, 25]. Exosomes released by glomerular endothelial cells, induced by high glucose, have been verified to activate mesangial cells, upregulate the expression of TGF-*β*1, activate the TGF-*β*1/Smad3 signaling cascade, and promote renal fibrogenesis [26]. The traditional Chinese medicine Tongxinluo was also verified to inhibit the secretion of TGF-*β*1 in exosomes derived from glomerular endothelial cells by high-glucose treatment and prevent the transfer of TGF-*β*1 from GECs to glomerular mesangial cells (GMCs) through exosomes, as well as GMCs TGF-*β*1/Smad3 signal pathway activation, thereby inhibiting the activation, proliferation, and matrix over-formation of GMCs [27]. Phagocytosis of exosomes by macrophages was of great significance to the immune response of diabetic nephropathy. Moreover, exosomes exist in various body fluids, and the biomarkers in the exosomes have the characteristics of convenient detection and slight damage.

Interestingly, tubular epithelial cell-generated exosomal miRNA-34a expressed higher in diabetic nephropathy Model-Exo in this study. MiRNA-34a is a critical regulatory factor in the mechanism of free fatty acid, which mediates impairment of pancreatic islet B-cell function and further participates in the pathogenesis of type 2 diabetes [28]. Jiang et al. [29] reported that the expression of miRNA-34a was verified to be significantly downregulated after stimulating the macrophage cell line RAW264.7 with LPS. Furthermore, miRNA-34a negatively regulated *PPARGC1A* in this study. *PPARGC1A* is an essential coactivator of the peroxide proliferation activation receptor. Recent studies have verified that diabetic nephropathy is related to mitochondrial dysfunction and increased endoplasmic reticulum stress [30, 31]. Interestingly, *PGC-1* played an essential role in mitochondrial biosynthesis, and excessive production of superoxide dismutase (ROS) produced by mitochondria was critical in the pathogenesis of diabetic nephropathy [32]. It was worth noting that *PPARGC1A* rescued macrophage polarization and renal tubular cell fibrosis in this study.

In recent years, more studies have confirmed that DN is an inflammatory disease [21]. The activation of the immune system and chronic inflammation are both involved in the pathogenesis of DN [33]. Macrophages are a type of immune cells with functional plasticity [34]. Under different stress conditions, the internal function and external phenotype may change. Xu et al. [35] used total glucosides of peony to treat streptozotocin-induced DN and confirmed that the degree of kidney damage and fibrosis in DN rats was related to the number of macrophages in the kidney. Moreover, the degree of macrophage infiltration was positively correlated with proteinuria, blood sugar, and blood creatinine levels [36]. Further study verified that these macrophages involved in kidney injury were of type M1, suggesting that the accumulation of type M1 macrophages in the kidney has become an essential feature of DN [37]. In addition, by reducing the expression of pro-inflammatory cytokines such as *MCP-1*, *IL-6*, *TNF-α*, *and IFN-γ* and increasing the expression of anti-inflammatory factors such as *IL-4* and *IL-10* and macrophages, it induced macrophages to transform into M2 type after entering the kidney and accumulated in the glomerulus and interstitium to prevent excessive inflammation [38]. Similarly results were also obtained in this study. The kidney exosomes of model mice caused the polarization of macrophages. Coculture of macrophages with model exosomes and TCMK-1 cells induced fibrosis of the renal tubular cells.

## 5. Conclusions

In conclusion, exosomal miRNA-34a of tubular epithelial cells promoted M1 macrophage activation in diabetic nephropathy via negatively regulating *PPARGC1A* expression. However, these results were only obtained from the diabetic nephropathy model, which needs to be verified in DN patients. Moreover, the pathogenesis of DN is complex. In future work, we will pay attention to more targets and establish a relationship network. Through this research, more possible effective targets for DN treatment will be provided.

## Data Availability

All data produced or analyzed during this study are included in the online shared database Figshare (https://figshare.com/) and can be accessed through the link 10.6084/m9 Figshare. 25389856.

## Supplementary Materials

Supplementary 1 Electron micrograph of multiple exosomes.

Supplementary 2 Sequence The relevant sequences of miRNA-34a inhibitor, inhibitor control, miRNA-34a mimics, mimics control, pc-PPARGC1A, and pc-NC.
