# Supplementary figures and images for: Renal Tissue-Derived Exosomal miRNA-34a in Diabetic Nephropathy Induces Renal Tubular Cell Fibrosis by Promoting the Polarization of M1 Macrophages
Source: IET Nanobiotechnol. 2024 Apr 17;2024:5702517. doi: 10.1049/2024/5702517 (PMC11095076; doi:10.1049/2024/5702517)

# Ctrl-Exo

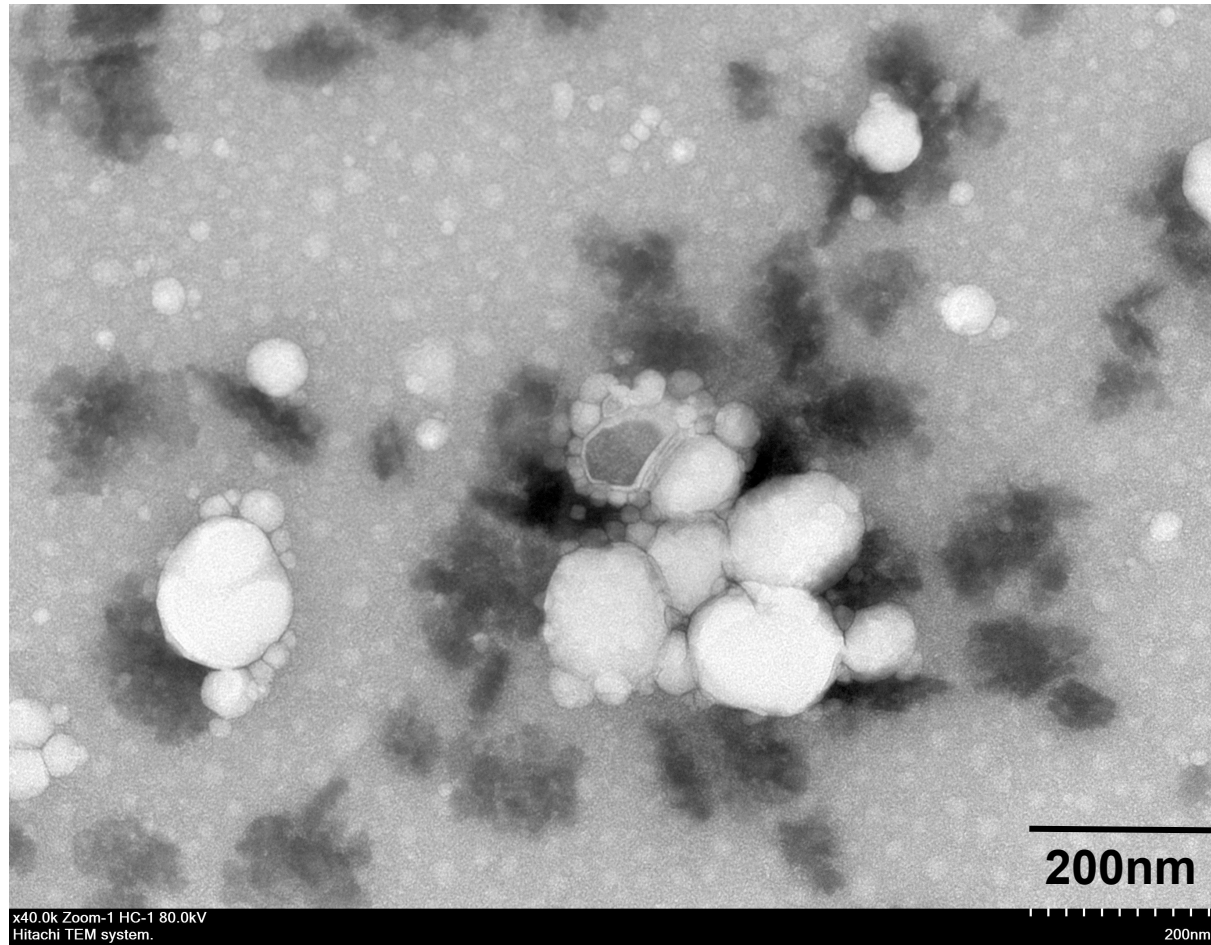

# Model-Exo

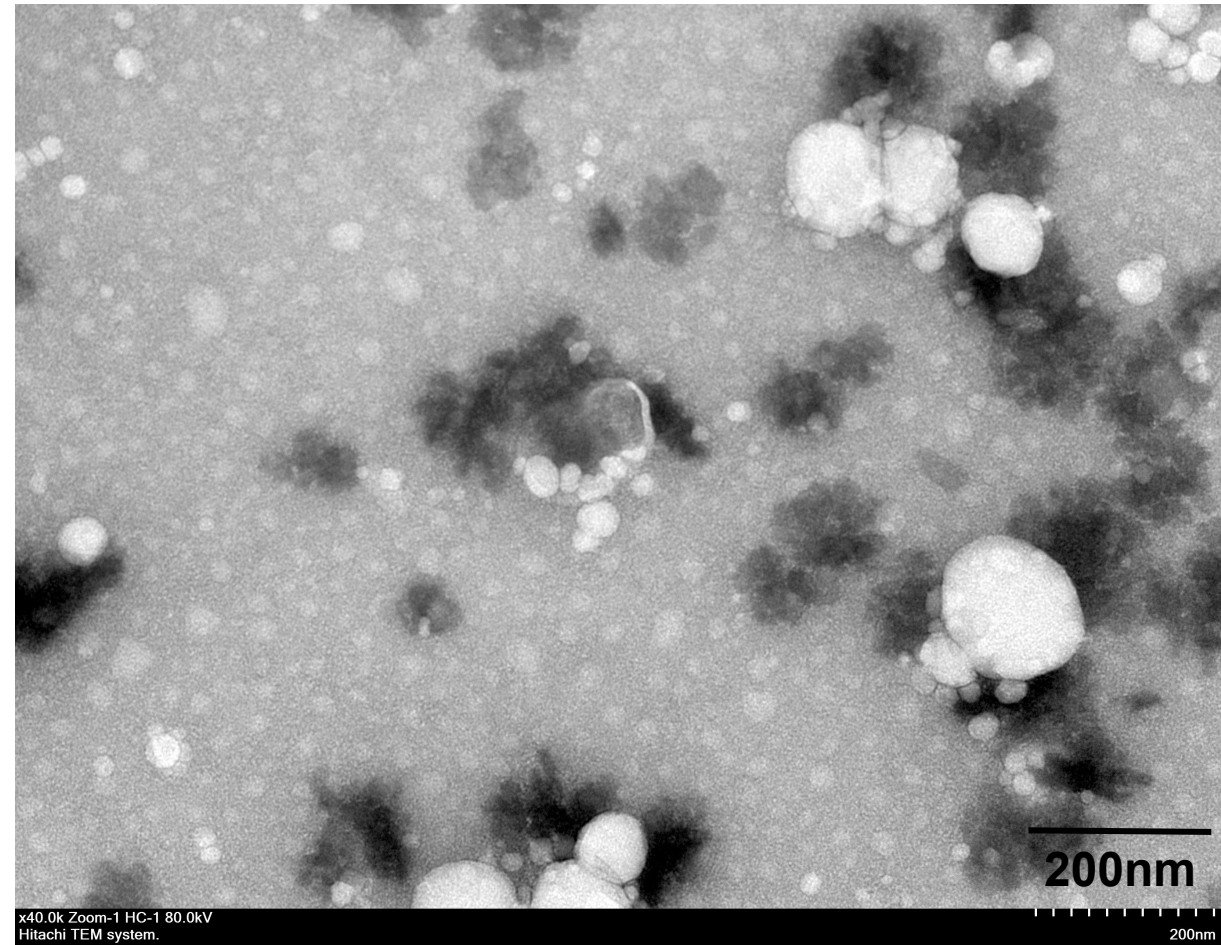

Supplement: Supplementary 1 — Electron micrograph of multiple exosomes. [file 5702517.f1.pdf]
